# 

**Published:** 1882-02

**Authors:** 


					﻿COLLABORATORS 3
ALF. L. LOOMIS, M. D.......New York.
FESSENDEN N. OTIS, M. D... .
F. H. BOSWORTH, Al. D......
J. L. LITTLE, Al. I)......
C. E. FRANCIS, D.D.S., M.D S....
A. J. C. SKENE, M. D. Brooklyn, N. Y.
C. A. MARVIN, D. D. S..
W.C. BARRETT, M. D., D. D.S.. .Buffalo, “
EDWIN T. DARBY, M.D..D.D.S... Phil., Pa.
P. S. WALES, M D., Surg. Gen’l, U. S. Navy.
SAM’LC. BUSEY, M. D....Wash’ll,	D. C.
H. F. CAMPBELL, M. D...Augusta,	Ga.
C. H. MASTIN, Al. D., L.L.D.Mobile, Ala.
J. J. CHISOLM. M. D.. ■..Baltimore, Md
C. F. BEVAN, M. D........
I.	E. ATKINSON, M.D...... "
H. McGUIRE, Al. D.......Richmond, Va.
F. P. PORCHER, Al. D.,..Charleston S. C.
J.	W. UNDERHILL, Al. D .... Cincinnati, O.
JAS. T. WHITTAKER, M. D..
J. RANSOHOFF, Al D„ F.R.C.S..	“
F. FORCHHEIA1ER, M. D.....	“	“
A. R. JACKSON, M. D...... Chicago, Ill
S. V. CLEVENGER, M.D........“
E. F. INGALS, M. D.....
J. H. CARSTENS, AI.D.....Detroit, Mich.
WILL YOU PLEASE SEND IN YOUR SUBSCRIPTION MONEY NO W
Personal.
This copy of the IN-
DEPENDENT PRACTI-
TIONER is sent to yon with
the hope that you will sub-
scribe.
It will not be continued
to any one, except PAID
UP Subscribers, UNLESS
so ordered.
Please let us hear from
you, and greatly oblige
Yours faithfully,
The Independent Practitioner Co.,
1099 BROADWAY, N. Y.
This Column For Sale to a First- Class
Advertiser.
Money is at Our Risk Only when sent by Post Office Money Order, Regis-
tered Letter, or Check made payable to the Independent Practitioner Co.,
1099 Broadway, N. Y. Please add 25 Cents for collecting checks payable
outside of New York City.
				

